# Toward a PSMA PET–mpMRI pathway for biopsy decision-making in men with suspected prostate cancer: interim results from the prospective BIOPSTAGE trial

**DOI:** 10.3389/fonc.2025.1720861

**Published:** 2026-01-05

**Authors:** Monica Celli, Roberta Gunelli, Fabio Ferroni, Matteo Costantini, Domenico Barone, Lorenzo Fantini, Eugenia Fragalà, Irene Marini, Valentina Di Iorio, Flavia Foca, Manuela Monti, Federica Matteucci, Paola Caroli

**Affiliations:** 1 Nuclear Medicine Unit, IRCCS Istituto Romagnolo per lo Studio dei Tumori (IRST) “Dino Amadori”, Meldola, Forlì-Cesena, Italy; 2 Department of Urology, AUSL Romagna, Morgagni-Pierantoni Hospital, Forlì, Forlì-Cesena, Italy; 3 Radiology Unit, IRCCS Istituto Romagnolo per lo Studio dei Tumori (IRST) “Dino Amadori”, Meldola, Forlì-Cesena, Italy; 4 Department of Pathology, AUSL Romagna, Morgagni-Pierantoni Hospital, Forlì, Forlì-Cesena, Italy; 5 Radiopharmacy, IRCCS Istituto Romagnolo per lo Studio dei Tumori (IRST) “Dino Amadori”, Meldola, Forlì-Cesena, Italy; 6 Unit of Biostatistics and Clinical Trials, IRCCS Istituto Romagnolo per lo Studio dei Tumori (IRST) “Dino Amadori”, Meldola, Forlì-Cesena, Italy

**Keywords:** biopsy guidance, clinically significant prostate cancer (csPCa), imaging biomarkers, ISUP grade group, lesion targeting, multiparametric MRI (mpMRI), prostate cancer, PSMA PET

## Abstract

**Introduction:**

The BIOPSTAGE trial investigates the added diagnostic value of combining prostate-specific membrane antigen positron emission tomography (PSMA PET) with multiparametric MRI (mpMRI) for non-invasive detection of clinically significant prostate cancer (csPCa). This interim analysis aimed to retrospectively optimize semiquantitative PSMA PET thresholds and evaluate potential biopsy reduction through integrated imaging.

**Methods:**

In this prospective, single-institution study, 96 men with suspected prostate cancer underwent mpMRI and PSMA PET prior to biopsy. csPCa was defined as ISUP grade group ≥2. Receiver operating characteristic (ROC) analysis identified optimal thresholds for SUVmax (≥9.1) and SUVratio (≥3.6). Diagnostic performance metrics (sensitivity, specificity, predictive values, and biopsy burden) were evaluated for mpMRI, PSMA PET, and their combination. Logistic regression and McNemar’s test assessed predictors and comparative accuracy.

**Results:**

mpMRI alone achieved a sensitivity of 93.8%, specificity of 54.6%, and an AUC of 0.74. Among the 74 lesions identified by mpMRI, 14 also met semiquantitative PET positivity criteria. An additional 11 PET-positive lesions were detected without corresponding mpMRI abnormalities. Another 60 lesions were mpMRI-positive but PET–negative and would not have been targeted in an integrated strategy. If biopsy had been guided exclusively by PSMA PET–positive lesions, 25 targets would have been sampled—representing an approximately 66.2% reduction compared to 74 lesions identified by mpMRI alone. This biopsy reduction was associated with significantly improved specificity (McNemar’s p < 0.001). Integrated imaging diagnostic accuracy was: sensitivity 85.7% (95% CI: 60.1–96.0%), specificity 86.3% (95% CI: 78.0–91.8%), PPV 48.0%, NPV 97.6%. Two small csPCa lesions missed by PSMA PET were PI-RADS 4, ISUP Grade Group 2. One csPCa lesion detected exclusively by PSMA PET was not visible on mpMRI (ISUP Grade Group 3).

**Conclusions:**

Integrating semiquantitative PSMA PET with mpMRI improves lesion-level specificity and was associated with a theoretical reduction of approximately two-thirds in the number of targeted biopsy cores, while maintaining high sensitivity. While PSMA PET enhances risk stratification, mpMRI remains essential—particularly for detecting small or PSMA-negative tumors. These interim findings support feasibility of a dual-imaging biopsy triage strategy, pending validation in the full BIOPSTAGE cohort and future multicenter trials.

## Introduction

Prostate cancer (PCa) is the most commonly diagnosed male malignancy in Europe and a leading cause of cancer−related morbidity and mortality (1). Early and accurate identification of clinically significant prostate cancer (csPCa) - defined as tumors with the potential for progression and harm if untreated - is essential for optimizing patient outcomes and minimizing overtreatment. Traditional diagnostic methods, such as serum prostate−specific antigen (PSA) testing and systematic transrectal ultrasound (TRUS)−guided biopsy, are limited by the overdiagnosis of indolent tumors and false negatives for aggressive disease (2). Consequently, there has been a shift toward imaging−based risk stratification. Multiparametric MRI (mpMRI) has become the standard first-line imaging modality for guiding prostate biopsies, owing to its high sensitivity and standardized interpretation via the Prostate Imaging Reporting and Data System (PI-RADS) (3). Nevertheless, mpMRI specificity remains suboptimal, particularly for PI-RADS 3 and PI-RADS 4 lesions, often leading to unnecessary biopsies and variable inter-reader agreement (4, 5). Prostate-specific membrane antigen (PSMA) positron emission tomography (PET) represents a complementary imaging approach, exploiting PSMA overexpression, which is particularly pronounced in higher-grade PCa (6). Although PSMA PET has demonstrated excellent performance in staging advanced disease, its role in primary csPCa diagnosis is still being investigated—largely due to limited standardization in uptake thresholds (7–10).

Refining semiquantitative PSMA PET metrics such as maximum standardized uptake value (SUVmax) and SUVratio is thus essential. This interim analysis of the prospective BIOPSTAGE trial aims to determine optimal semiquantitative PET thresholds for SUVmax and SUVratio that effectively discriminate csPCa, and to evaluate whether enrollment should continue. We assessed the integration of semiquantitative PSMA PET with mpMRI to enhance csPCa detection, while also analyzing the influence of 5-alpha reductase inhibitors (5ARI) on benign prostatic PSMA uptake.

Our goal is to improve biopsy targeting by selecting only the most significant imaging findings—thereby reducing unnecessary sampling without compromising sensitivity. A growing body of evidence supports the complementary value of mpMRI and PSMA PET for prostate cancer risk stratification (8, 11–13). In parallel, recent studies have emphasized the need for standardized acquisition protocols and quantitative assessment to ensure reproducibility (12, 14–17).

Building on this work, our interim results contribute robust preliminary evidence for the use of combined PSMA PET and mpMRI imaging in csPCa triage, patient selection, and biopsy planning (18–20). Notably, this study is the first to report *in vivo* the influence of 5ARI therapy on benign PSMA uptake, offering new insights into hormonal modulation of imaging biomarkers (16, 21). Ultimately, our findings aim to inform the development of an integrated PET–mpMRI pathway for biopsy decision-making in men with suspected clinically significant prostate cancer.

## Materials and methods

### Study design and participants

The BIOPSTAGE trial (ClinicalTrials.gov Identifier: NCT03465579) is a prospective, single-institution interventional diagnostic study conducted at the IRCCS Istituto Romagnolo per lo Studio dei Tumori (IRST) “Dino Amadori,” Meldola, Italy. The trial is part of the Comprehensive Cancer Center Romagna Network (CCCRN). The study protocol was approved by the local ethics committee (protocol code IRST185.05).

This interim analysis includes the first 96 men consecutively enrolled by urologists from the Department of Urology, Forlì Hospital (AUSL Romagna), based on clinical or biochemical suspicion of prostate cancer (PCa). Eligibility criteria included one or more of the following: persistently elevated serum PSA, reduced free-to-total PSA ratio (within the 4–10 ng/mL range), PSA density >0.1 ng/mL/cc, abnormal digital rectal examination (DRE), or histologic evidence of premalignant changes from a previous biopsy. Both biopsy-naïve men and those with a negative transrectal ultrasound (TRUS)-guided biopsy performed ≥12 months prior to enrollment were eligible.

### Imaging protocols

mpMRI and PSMA PET were performed within a median interval of 9 days (IQR 6–14; range 0–35 days), always before biopsy and without any intervening treatment modification.

#### Multiparametric MRI

All patients underwent pre-biopsy mpMRI on a 3.0 Tesla Ingenia^®^ MR scanner (Philips Healthcare, The Netherlands), including T2-weighted, diffusion-weighted imaging (DWI) with ADC maps, and dynamic contrast-enhanced (DCE) sequences. Two uroradiologists with over five years of experience interpreted the scans independently and were blinded to PSMA PET and clinical data. Lesions were scored using the Prostate Imaging Reporting and Data System version 2.0 (PI-RADS v2.0). Up to two lesions per patient with the highest PI-RADS scores—or the largest if scores were equal—were selected for biopsy targeting.

For inter-observer analyses, each reader also assigned an exam-level binary label (positive/negative). mpMRI was considered exam-positive if at least one lesion with PI-RADS ≥3 was present; otherwise the exam was negative.

#### PSMA PET

[^68^Ga]Ga-PSMA-11 PET/CT was performed following a ≥4-hour fasting period. Patients received a dose of 2.0 MBq/kg of [^68^Ga]Ga-PSMA-11, with image acquisition 60 minutes post-injection using a Biograph mCT^®^ PET/CT scanner (Siemens Healthineers, Germany). Whole-body images from vertex to mid-thigh were acquired, and low-dose non-contrast CT was used for attenuation correction. Two experienced nuclear medicine physicians independently identified all PSMA-avid foci, blinded to mpMRI and clinical data. A maximum of two lesions per patient with the highest uptake were recorded.

For inter-observer analyses, each reader also assigned an exam-level binary label (positive/negative). PET was considered exam-positive if ≥1 visually PSMA-avid focus suspicious for prostate cancer was present; otherwise the exam was negative.

### Reader agreement analysis (exam-level)

Two experienced readers independently and blindly reviewed all examinations. Inter-observer agreement between the two independent exam-level labels (positive/negative) was quantified using Gwet’s AC1 (primary metric) with 95% confidence intervals obtained by percentile bootstrap (3,000 resamples). As complementary indices, we report Cohen’s κ, overall percent agreement, and positive/negative percent agreement (PPA/NPA), each with 95% bootstrap CIs. Analyses were performed at the exam level with pairwise exclusion of missing or non-binary entries. No post-hoc consensus was applied in primary analyses to avoid artificially reducing inter-reader variability.

When a single final exam label was required for descriptive summaries, we used a pre-specified “any-positive” rule (exam positive if ≥1 reader was positive); a stricter “both-positive” rule was explored as a sensitivity analysis.

### Image fusion and biopsy procedures

mpMRI and PSMA PET images were co-registered using Syngo.via software (Siemens Healthineers) to align lesions within a standardized 12-segment prostate map. Targeted biopsy was performed for all PI-RADS ≥3 lesions. PET-avid lesions overlapping with mpMRI targets were sampled as part of standard image-guided biopsy. PET-avid lesions without a corresponding mpMRI target (‘PET-only’) were not targeted with separate fusion-guided cores but were sampled only within the standard 12-core TRUS systematic biopsy. This design was chosen to reflect current clinical practice and to avoid adding extra targeted cores in this interim phase.

### Histopathological assessment

Two genitourinary pathologists reviewed all biopsy specimens. Clinically significant PCa (csPCa) was defined as ISUP Grade Group ≥2. Clinically insignificant PCa (ciPCa) and benign findings (e.g., inflammation, BPH) were also recorded.

### Statistical analysis

**Figure 1:**
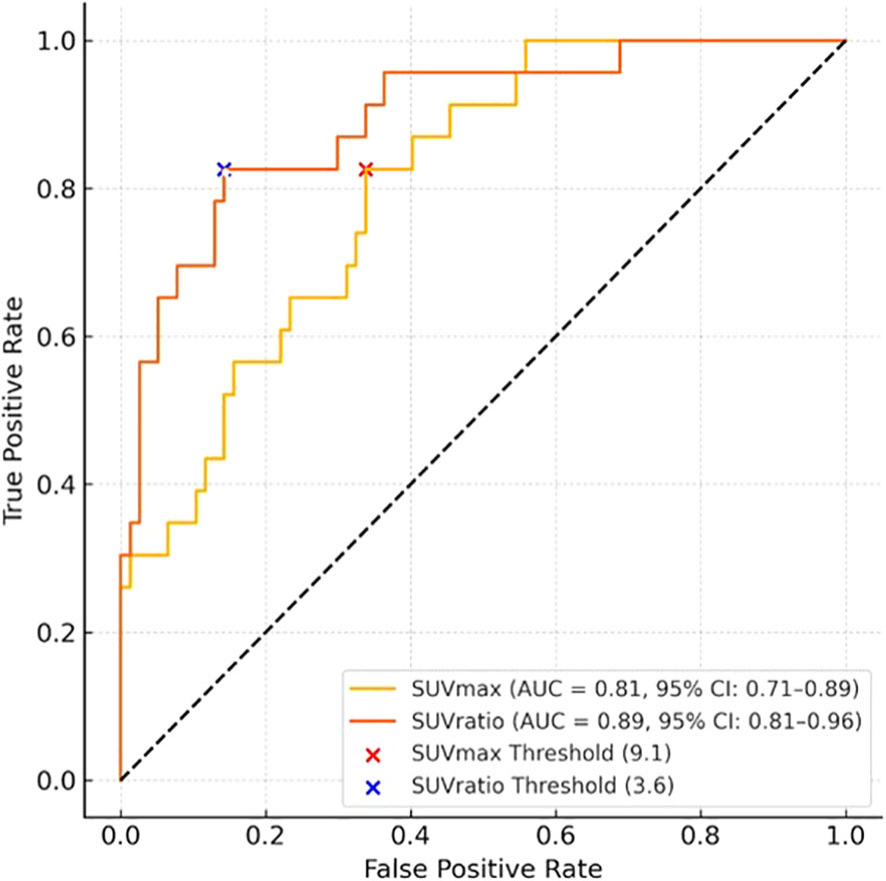
Receiver operating characteristic (ROC) curves for SUVmax and SUVratio in detecting clinically significant prostate cancer (csPCa). The area under the curve (AUC) and 95% confidence intervals (CIs) were estimated using 2,000 bootstrap replicates. Discrimination was strong for both PET parameters: AUC for SUVmax = 0.82 (95% CI: 0.71–0.89) and for SUVratio = 0.89 (95% CI: 0.81–0.96). The pre-specified Youden-optimized thresholds (SUVmax ≥ 9.1; SUVratio ≥ 3.6) are indicated by red and blue markers, respectively. Additional points along each curve represent alternative operating thresholds to guide assessment of test performance under varying clinical risk tolerance.

PSMA PET SUVmax and SUVratio (lesion SUVmax divided by background SUVmean) were evaluated for each lesion. Threshold selection for SUVmax and SUVratio (lesion-to-background ratio) was pre-specified and based on receiver operating characteristic (ROC) analysis. The optimal cut-point for each metric was determined using the Youden Index (J = sensitivity + specificity − 1), representing the point that best balanced sensitivity and specificity for detecting clinically significant prostate cancer. Uncertainty for AUCs and derived performance metrics was quantified through percentile-bootstrap 95% confidence intervals (B = 3,000, seed = 17). SUVratio was introduced to improve reproducibility and cross-platform comparability by partially normalizing for inter-scanner variability and patient-specific background uptake. ROC curves with AUCs and the selected thresholds are shown in **Figure 1** (Supplementary Figure S1).

Diagnostic accuracy metrics—sensitivity, specificity, positive predictive value (PPV), negative predictive value (NPV), and area under the ROC curve (AUC)—were calculated for mpMRI, PSMA PET, and the integrated strategy. McNemar’s test was used to compare biopsy target counts and specificity between mpMRI and integrated imaging strategies. Logistic regression was performed to evaluate predictors of csPCa and PET positivity. A two-tailed p-value <0.05 was considered statistically significant.

Inter-observer agreement for PSMA PET was quantified using Gwet’s AC1 as the primary metric, complemented by observed agreement (P_o_), positive/negative agreement, Cohen’s κ, prevalence-adjusted bias-adjusted κ (PABAK), and McNemar’s test for asymmetry of discordance. Uncertainty was estimated using percentile-bootstrap 95% confidence intervals (B = 3000, seed = 17).

Analyses were performed using Stata version 15.1 (StataCorp, TX, USA).

## Results

### Study population and baseline characteristics

Out of 111 men screened, 96 completed all study procedures and were included in this interim analysis. The median age was 64 years (interquartile range [IQR]: 59.8–67.2), the median PSA level was 6.0 ng/mL (IQR: 4.1–8.0), and the median prostate volume was 70.0 cc (IQR: 54.0–90.5). (**Table 1**). Histopathological evaluation identified clinically significant prostate cancer (csPCa; ISUP grade group ≥2) in 14 subjects (14.6%) and clinically insignificant PCa (ciPCa) in 13 subjects (13.5%). In total, 16 csPCa-positive lesions were identified across these 14 men, as two of them harbored more than one significant lesion. Among csPCa lesions, ISUP grade 2 was most frequent (8 cases), followed by ISUP grade 4 (3 cases), ISUP grade 5 (3 cases), and ISUP grade 3 (2 cases). PSA density was the only significant predictor of csPCa, with an odds ratio (OR) of 3.12 (95% CI: 1.21–5.76; p = 0.031).

**Table 1:** Summary of demographic and clinical characteristics for the study cohort (n = 96).

Metric	Median (IQR) or frequency (%)
Age (years)	64.0 (59.8–67.2)
Serum PSA (ng/mL)	6.00 (4.10–8.03)
*PSA ≥ 10 ng/mL*	18 (18.8%)
*PSA < 10 ng/mL*	78 (81.2%)
PSA Free-to-Total Ratio (%)	13.75 (10.03–16.18)
*PSA Free-to-Total Ratio (%) ≤ 15%*	50 (64.1%)
*PSA Free-to-Total Ratio (%) > 15%*	28 (35.9%)
Prostate Volume (cc)	70.0 (54.0–90.5)
PSA Density (ng/mL/cm³)	0.09 (0.06–0.12)
Prior Biopsy Performed	80 (83.3%)
HGPIN/ASAP on Prior Biopsy	30 (37.5% of those biopsied)
csPCa Positive	14 (14.6%)
csPCa Negative	82 (85.4%)

### Diagnostic accuracy of mpMRI

Multiparametric MRI identified 74 lesions with PI-RADS scores ≥3 in 54 patients. Among these, 31 were classified as PI-RADS 3 and yielded no csPCa, while PI-RADS 4 (n = 32) and PI-RADS 5 (n = 11) lesions had csPCa detection rates of 25.0% (n=8) and 63.6% (n=7), respectively. At a threshold of PI-RADS ≥3, mpMRI achieved a sensitivity of 93.8% (95% CI: 89.0–98.0%), a specificity of 54.6% (95% CI: 45.0–64.0%), a positive predictive value (PPV) of 20.3%, and a negative predictive value (NPV) of 98.6%. The area under the receiver operating characteristic curve (AUC) was 0.74.

### ROC transparency and diagnostic accuracy of PSMA PET

ROC analysis supported both SUVmax and SUVratio as strong discriminators of clinically significant prostate cancer, with AUCs of 0.82 (95% CI 0.71–0.89) and 0.89 (95% CI 0.81–0.96), respectively. The Youden-optimized thresholds**—**SUVmax ≥ 9.1 and SUVratio ≥ 3.6—are marked in **Figure 1** (Supplementary Figure S1), alongside alternative candidate thresholds for visual comparison; corresponding performance metrics are summarized in **Table 2** (Supplementary Table S1). Applying these thresholds, 25 lesions (in 21 patients) were classified as PSMA PET–positive. On this basis, PSMA PET achieved sensitivity 85.7% (95% CI 60.1–96.0%), specificity 86.3% (95% CI 78.0–91.8%), PPV 48.0% (95% CI 30.0–66.5%), and NPV 97.6% (95% CI 91.7–99.3%). Two small csPCa lesions (5 mm and 8 mm; PI-RADS 4) were not detected by PSMA PET (SUVmax 4.6 and 5.8; SUVratio 2.4 and 1.9), whereas one csPCa lesion (ISUP Grade Group 3) was identified by PSMA PET alone and not visible on mpMRI. In multivariable analysis, PSA density remained an independent predictor of PSMA PET positivity (OR 6.35; 95% CI 1.82–10.88; p=0.006).

**Table 2:** Comparison of logistic regression results for csPCa prediction and PSMA PET positivity.

Variable	OR csPCa prediction	95% CI	p-value	OR PSMA PET positivity	95% CI	P-value
PSA Density	3.12	1.21 – 5.76	0.031	6.35	1.82 – 10.88	0.006
Age	1.02	0.96 – 1.08	0.517	1.0028	-0.061 – 0.067	0.932
Prior Negative Biopsy	0.81	0.34 – 1.94	0.635	1.47	-0.957 – 1.737	0.570

OR, Odds Ratio; CI, Confidence Interval.

### Inter-observer agreement

Inter-reader agreement was high for both modalities. PSMA PET (N=96): overall agreement 94.8%; Gwet’s AC1 0.913 (95% CI 0.826–0.983); Cohen’s κ 0.870 (95% CI 0.742–0.972); PPA 96.4% (95% CI 92.9–99.3); NPA 90.6% (95% CI 80.9–98.0). mpMRI (N=96): overall agreement 91.7%; AC1 0.836 (95% CI 0.716–0.938); κ 0.831 (95% CI 0.706–0.936); PPA 92.6% (95% CI 86.7–97.2); NPA 90.5% (95% CI 82.8–96.4). Analyses were performed at the exam level with pairwise exclusion of non-binary/missing entries; no post-hoc consensus was applied.

### Integrated imaging performance and biopsy reduction

**Figure 2:**
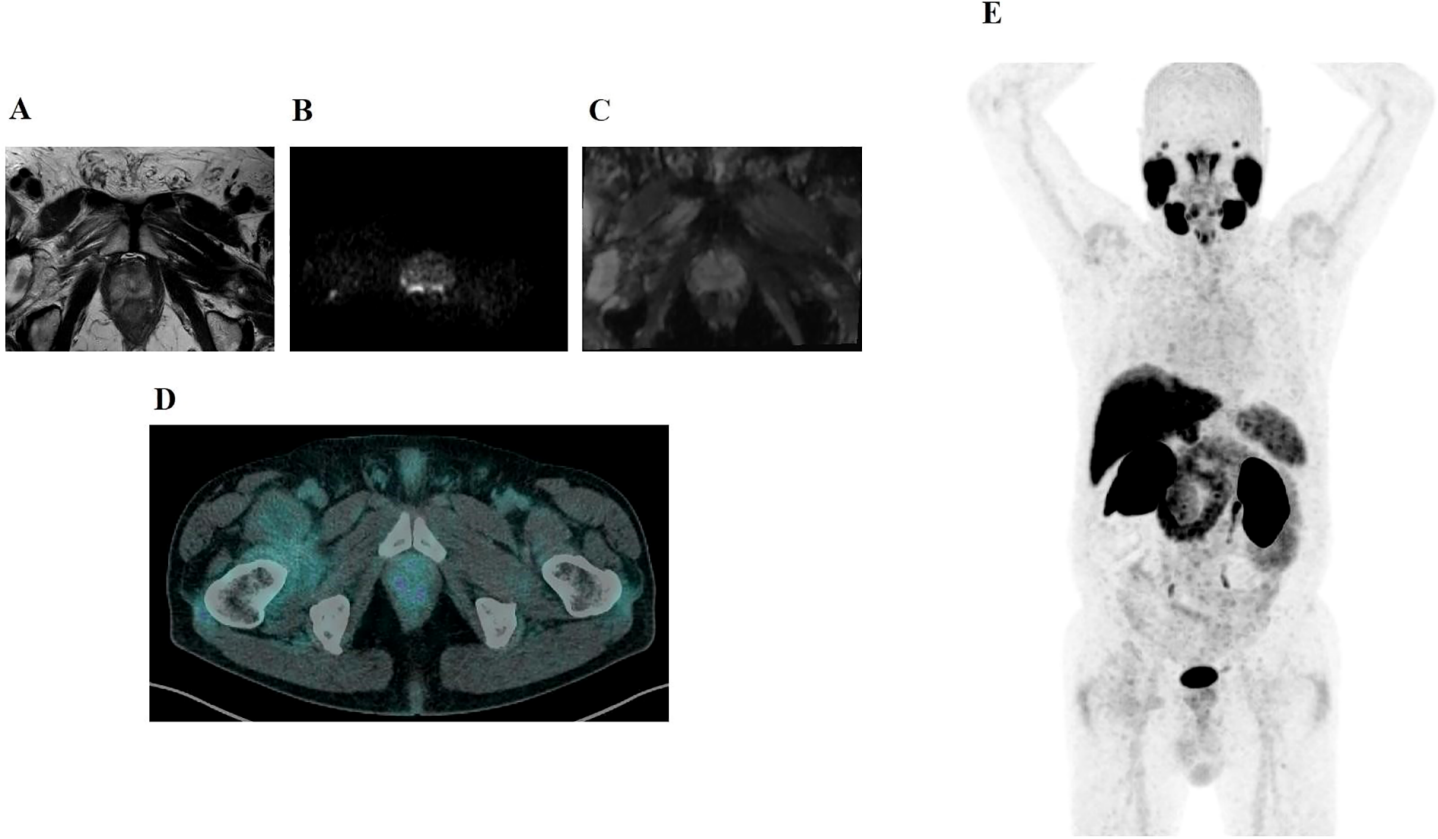
A 76-year-old man with a serum PSA of 3.7 ng/mL and a PSA density of 0.06 ng/mL/cc. BIOPSTAGE mpMRI identified an 8 mm PI-RADS 3 lesion in the apical posteromedial right peripheral zone. Axial T2-weighted MRI **(A)** shows no significant abnormalities, while DWI **(B)** demonstrates hyperintensity, and the ADC map **(C)** reveals mild hypointensity. However, axial PSMA PET **(D)** and MIP PSMA PET **(E)** show no abnormal tracer uptake. MRI-targeted biopsy and systematic biopsy confirmed the absence of csPCa, emphasizing the role of PSMA PET in avoiding overdiagnosis and unnecessary intervention for PI-RADS 3 lesions.

Among the 74 mpMRI-positive lesions (PI-RADS ≥3), 14 (18.9%) were also semiquantitatively PSMA PET–positive (concordant positivity of PSMA PET and mpMRI), while 60 lesions (81.1%) were PSMA PET–negative. (**Figure 2**).

An additional 11 lesions were identified as PSMA PET–positive without corresponding mpMRI abnormalities (PET-only). PSMA PET positivity increased with higher PI-RADS category: 9.7% (3/31) in PI-RADS 3, 12.5% (4/32) in PI-RADS 4, and 63.6% (7/11) in PI-RADS 5. Using the integrated PSMA PET–mpMRI strategy, in which only semiquantitatively PSMA PET–positive lesions (SUVmax ≥ 9.1 or SUVratio ≥ 3.6) were selected for biopsy, 25 lesions would have been sampled: 14 concordant PSMA PET and MRI positive lesions and 11 PET-only positive lesions. This corresponds to a theoretical 66.2% reduction in targeted biopsies compared to the 74 mpMRI-based lesions. McNemar’s test confirmed a statistically significant improvement in specificity with the integrated approach (from 54.6% to 86.3%; p < 0.001).

**Figure 3:**
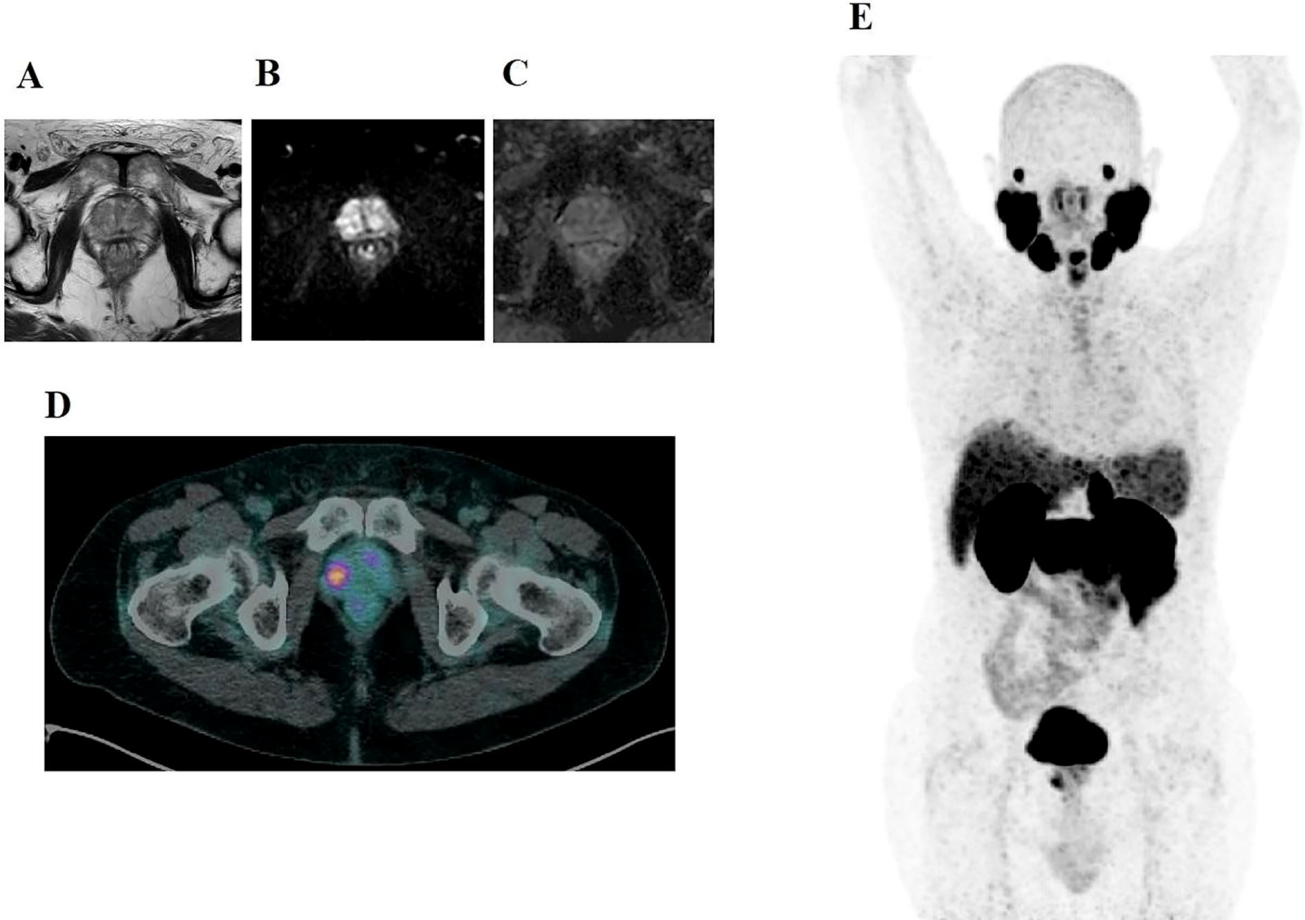
A 72-year-old man with a serum PSA of 4.5 ng/mL and a PSA density of 0.10 ng/mL/cc. BIOPSTAGE mpMRI was negative for suspicious findings (PI-RADS <3), while PSMA PET detected a focal uptake in the right anterior apex of the prostate. Axial T2-weighted MRI **(A)** shows no significant abnormalities. Axial diffusion-weighted imaging (DWI) **(B)** reveals no focal hyperintense signals, and the apparent diffusion coefficient (ADC) map **(C)** shows no restricted diffusion. In contrast, axial PSMA PET **(D)** and MIP PSMA PET **(E)** demonstrate focal uptake in the right anterior apex (SUVmax: 8.1; SUVratio: 3.6). Biopsy confirmed an ISUP 3 prostate cancer focus in the PSMA-positive region and minor ISUP 1 bilateral foci. This case highlights the complementary role of PSMA PET in detecting csPCa lesions missed by mpMRI.

The integrated strategy yielded a sensitivity of 85.7% (95% CI: 60.1–96.0%), specificity of 86.3% (95% CI: 78.0–91.8%), PPV of 48.0% (95% CI: 30.0–66.5%), and NPV of 97.6% (95% CI: 91.7–99.3%). Importantly, csPCa was present in 64.3% (9/14) of concordant PSMA PET and MRI positive lesions but in only 9.1% (1/11) of PET-only lesions, indicating higher diagnostic yield in concordant findings. (**Figure 3**).

### Detection of clinically insignificant prostate cancer

ciPCa was detected in 13 participants (13.5%): 5 cases (38.5%) via MRI-targeted biopsy and 8 (61.5%) via systematic biopsy. None of the ciPCa cases were detected by the integrated imaging strategy, which could have prevented their overtreatment.

### Influence of 5α-reductase inhibitor therapy on PSMA uptake

Evaluation of background PSMA uptake in benign prostate tissue revealed a median SUVmean of 2.20 (95% CI: 2.00–2.40) for atrophy/inflammation and 2.40 (95% CI: 2.20–2.70) for benign prostatic hyperplasia. Patients receiving 5ARI therapy showed significantly higher median SUVmean values (2.40 vs. 2.20 in non-users; p = 0.032), suggesting that 5ARI use may increase PSMA expression in benign tissue and potentially influence PET interpretation.

## Discussion

This interim analysis suggests that integrating semiquantitative PSMA PET with mpMRI can refine the diagnostic workup for csPCa while reducing unnecessary biopsies. While mpMRI offers excellent sensitivity, its relatively low specificity—particularly for PI-RADS 3 and PI-RADS 4 lesions—contributes to a high rate of false positives and excessive biopsy targeting (1, 4). The application of optimized semiquantitative PSMA PET thresholds (SUVmax ≥ 9.1 and SUVratio ≥ 3.6) markedly improved specificity. When these thresholds were applied in a strategy where only PSMA PET–positive lesions were considered for biopsy, specificity increased from 54.6% with mpMRI alone to 86.3% with the integrated approach. In parallel, the number of biopsy targets was reduced from 74 (mpMRI-based) to 25 (PSMA PET–filtered), corresponding to a 66.2% theoretical reduction. This gain in diagnostic efficiency was statistically significant, as confirmed by McNemar’s test (p < 0.001). While the thresholds were pre-specified and internally validated through bootstrapping, absolute SUV cut-points may vary across centers depending on scanner type, reconstruction algorithms, and acquisition timing. Reporting both an absolute metric (SUVmax) and a normalized metric (SUVratio) enhances portability by accounting for inter-system variability. Nevertheless, external validation and harmonization—such as cross-calibration or EARL-like standardization—will be essential to ensure reproducibility in multicenter settings. The inclusion of ROC curves and AUCs (**Figure 1**) makes the operating points explicit and allows assessment of alternative threshold choices in other contexts.

Our findings should be interpreted in the context of MRI-first pathways such as PROMIS and PRECISION, which established mpMRI as a triage test before biopsy. In these trials (22, 23), mpMRI showed high sensitivity and negative predictive value for csPCa and allowed MRI-targeted biopsy to increase csPCa detection while reducing overdiagnosis of clinically insignificant cancer and avoiding biopsy in approximately one quarter of men. However, mpMRI alone still yields a substantial number of false-positive PI-RADS 3–4 lesions and misses a proportion of csPCa, motivating the search for complementary imaging biomarkers.

Several studies have evaluated the addition of PSMA PET to mpMRI or the use of combined PET/MRI scanners in the primary diagnostic setting. Simultaneous 68Ga-PSMA PET/MRI has been shown to improve intraprostatic tumor localization compared with mpMRI or PET alone, particularly for index lesions. Other groups have reported that combining mpMRI with PSMA PET improves detection of csPCa compared with systematic biopsy and may reduce false negatives compared with MRI alone. Sharma et al. (24) further demonstrated that, in men with PI-RADS 4/5 lesions, a combined SUVmax and PI-RADS threshold could predict malignancy with high specificity, supporting the concept of a biopsy-sparing pathway in highly selected patients.

Our results are consistent with these observations in showing that PSMA PET adds complementary information to mpMRI, but our contribution is to define ROC-derived semiquantitative thresholds that can be used to filter mpMRI-positive lesions and to quantify the theoretical impact on biopsy workload.

However, it should be acknowledged that the 9.1% csPCa detection rate observed in PET-only lesions in our cohort is probably conservative. PET-only foci were not subjected to dedicated PET-targeted biopsy but were sampled only within the standard 12-core TRUS scheme, which is known to under-detect anterior and small-volume tumors. As a result, some PSMA PET–positive/MRI-negative lesions that are classified as benign in our dataset may in fact harbor csPCa that was not captured by systematic cores. This verification bias may contribute to an underestimation of the diagnostic yield of PET for MRI-occult disease and, at the same time, influence apparent PET–mpMRI concordance.

Although PSMA uptake has been reported in some benign intraprostatic conditions, focal uptake clearly above background is highly specific for malignancy in most series, and we did not identify a consistent benign histologic substrate that could explain PET-only/biopsy-negative lesions in our cohort. We therefore consider biopsy under-sampling, rather than a high prevalence of benign PSMA-avid nodules, to be the main explanation for the low observed csPCa rate in PET-only lesions.

These findings should also be interpreted in light of previous PSMA PET-guided biopsy and PET–mpMRI studies. Kumar et al. (25) reported high cancer yields when PSMA-avid intraprostatic foci were directly targeted with robotic PET/CT-guided biopsies while Sharma et al. (24) further demonstrated that, in men with PI-RADS 4/5 lesions, a combined SUVmax and PI-RADS threshold could predict malignancy with high specificity, supporting the concept of a biopsy-sparing pathway in highly selected patients. showed that combining PSMA PET-CT with mpMRI in highly selected patients yielded high sensitivity and specificity for localized disease. In contrast to these studies, our PET-only subgroup lacked an mpMRI correlate, was verified predominantly by systematic rather than PET-targeted sampling, and included lesions fulfilling relatively inclusive PET positivity criteria. Differences in case mix, sampling strategy, and positivity thresholds therefore likely account for the lower apparent csPCa detection rate in PET-only lesions in our study.

Overall, our findings remain consistent with the concept that PSMA PET provides complementary information to mpMRI. Our contribution is to define ROC-derived semiquantitative thresholds that can be used to filter mpMRI-positive lesions and to quantify the theoretical impact on biopsy workload.

Inter-reader reliability at the exam level was high—Gwet’s AC1 was 0.913 for PSMA PET and 0.836 for mpMRI (3,000-bootstrap 95% CIs 0.826–0.983 and 0.716–0.938, respectively)—supporting the robustness of our qualitative labels and the thresholds derived from them. Logistic regression analysis supported the relevance of PSMA PET as a risk stratification tool. Lesions with higher ISUP grade (particularly Grade Group ≥2) were more likely to exhibit high PSMA uptake, reinforcing the link between PSMA expression and tumor aggressiveness (6, 11, 12, 26). Notably, PSA density emerged as a statistically significant predictor of PSMA PET positivity (OR 6.35; 95% CI 1.82–10.88; p=0.006), reinforcing its role as a valuable biomarker in prostate cancer imaging and supporting its inclusion in future risk stratification models (16). Two small csPCa foci in patients with low PSA density were not detected by PSMA PET but were captured by mpMRI, underscoring mpMRI critical role in detecting low-PSMA-expressing lesions. Conversely, PSMA PET alone identified one csPCa lesion that was not visible on mpMRI, highlighting its potential complementary value. Notably, the frequency of csPCa was substantially higher in lesions that were positive on both PSMA PET and mpMRI (64.3%) compared to PSMA PET–only lesions (9.1%), supporting the value of combining modalities for triage refinement. Additionally, the integrated strategy correctly excluded all cases of ciPCa that would otherwise have been biopsied using mpMRI alone, potentially mitigating overdiagnosis and overtreatment.

The observed increase in benign prostatic SUVmean among patients receiving 5α-reductase inhibitors was modest in absolute terms (median 2.4 vs 2.2) and is unlikely to affect lesion-level classification when lesion SUVmax values are well above the diagnostic threshold (SUVmax ≥ 9.1). However, this small shift in background uptake may influence SUV-based ratios for lesions close to the cut-off and suggests that the hormonal milieu can modulate PSMA expression in benign tissue. Accordingly, this result should be considered exploratory and hypothesis-generating, and future studies should further evaluate whether 5ARI exposure warrants tailored SUVratio thresholds (17).

While these interim results are promising, limitations include the single-center setting and modest cohort size. Nevertheless, the analytic rigor—incorporating ROC analysis, logistic modeling, and paired comparisons—provides a strong foundation for continued enrollment. Future work should focus on validating semiquantitative thresholds in larger, multicenter settings and on developing harmonized protocols to address variability in SUV quantification across imaging platforms. The final analysis of the BIOPSTAGE trial is anticipated to provide additional insights into threshold robustness and the real-world clinical applicability of this integrated imaging strategy.

Beyond diagnostic performance, the implementation of a PET–mpMRI pathway must consider cost, radiation exposure, and access. PSMA PET/CT remains less widely available and more expensive than mpMRI in many regions, and our integrated strategy is therefore unlikely to represent a first-line test for all men with suspected PCa. Instead, it may be most appropriate as a second-line or problem-solving tool in patients with persistent clinical suspicion after equivocal or negative mpMRI, or in centers where PSMA PET is already used for staging. The radiation dose of a single 68Ga-PSMA PET/CT scan is modest but not negligible, and any biopsy-sparing benefit must be weighed against this exposure. Ultimately, formal cost-effectiveness analyses and health-economic modelling will be required before routine clinical adoption can be recommended.

In summary, this interim analysis of the prospective BIOPSTAGE trial suggests that integrating semiquantitative PSMA PET with mpMRI can increase lesion-level specificity and may substantially reduce the number of targeted biopsy cores required, without compromising sensitivity for csPCa. However, these findings are based on a single-center cohort with only 14 patients harboring csPCa, and the estimated two-thirds reduction in targeted biopsies should be considered theoretical. The results are therefore hypothesis-generating rather than practice-changing and require confirmation in the final BIOPSTAGE cohort and in larger multicenter studies before any transformation of current diagnostic pathways can be recommended.

## Limitations

An important limitation of our study is the potential for verification bias. PET-only lesions were not subjected to dedicated PET-targeted biopsy, but were sampled only as part of the 12-core TRUS systematic biopsy, which is known to under-detect anterior and anterior-apical tumors. Consequently, the observed 9.1% csPCa rate in PET-only lesions likely underestimates the true prevalence of clinically significant disease in this subgroup and may lead to an underestimation of PSMA PET sensitivity for MRI-occult lesions. This design contrasts with PET-guided biopsy studies in which PSMA-avid lesions are directly targeted, yielding substantially higher csPCa rates and diagnostic yield. Our integrated PET–mpMRI results should therefore be interpreted as conservative and hypothesis-generating. Another potential source of bias is the interval between mpMRI and PSMA PET acquisition. In our protocol, the two examinations were performed within a median of 9 days (IQR 6–14; range 0–35 days) and always before biopsy, without any intervening treatment modifications. Given the relatively slow natural history of localized prostate cancer, we consider it unlikely that this short time gap substantially altered lesion status; however, minor temporal changes in tumor biology or PSA cannot be completely excluded.

## Conclusions

This interim analysis of the BIOPSTAGE trial indicates that integrating semiquantitative PSMA PET with mpMRI can improve lesion-level specificity and may allow a more selective biopsy strategy in men with suspected clinically significant prostate cancer. While mpMRI alone remains an excellent high-sensitivity triage test, ROC-derived PSMA PET metrics appear promising for refining biopsy selection among mpMRI-positive lesions. Lesions that are positive on both modalities carried the highest risk of csPCa, supporting the value of concordant imaging for triage. Conversely, the small number of csPCa foci missed by PSMA PET but detected by mpMRI underscores that mpMRI cannot be omitted. Given the limited sample size and single-center design, these findings should be viewed as preliminary. Validation in the completed BIOPSTAGE cohort and in external multicenter series, as well as formal cost-effectiveness analyses, will be crucial before PET–mpMRI triage can be integrated into routine clinical practice.

## Data Availability

The raw data supporting the conclusions of this article will be made available by the authors, without undue reservation.
